# Use of MALDI-TOF VITEK MS for rapid and efficient identification of KPC-type carbapenemases in Enterobacterales carrying the Tn4401a transposon

**DOI:** 10.1007/s10096-025-05097-6

**Published:** 2025-04-03

**Authors:** Aleyda M. Montaño, Carlos Robledo, Julián C. Galvis-Ayala, J. Natalia Jimenez, Romain Brunel, Jaime Robledo

**Affiliations:** 1 Laboratorio Medico de Referencia, Medellín, Colombia; 2 Grupo de Investigación en Microbiología Básica y Aplicada, Escuela de Microbiología, MICROBA, Universidad de Antioquia, Medellín, Colombia; 3 Antimicrobial Stewardship Centers of Excellence, Laboratorio Medico de Referencia - Clinica El Rosario– bioMérieux, Medellin, Colombia; 4 bioMérieux Colombia SAS, Bogotá, Colombia; 5 Corporación para Investigaciones Biológicas, Medellín, Colombia

**Keywords:** Maldi Tof, KPC-type carbapenemase, *bla*_*KPC*_ gene, pKpQIL plasmid, Tn4401a transposon

## Abstract

**Purpose:**

To determine diagnostic validity of MALDI-TOF MS (VITEK MS) system for detecting Klebsiella pneumoniae carbapenemases (KPC)-*type* carbapenemases by identifying the 11,109 Da peak in the mass spectrum generated for species identification as compared to RAPIDEC^®^ CARBA NP, and the modified carbapenemase inactivation method (mCIM) and the EDTA-modified carbapenem inactivation method (eCIM) in a collection of isolates previously characterized as KPC positive or negative.

**Methods:**

210 Enterobacterales clinical strains previously characterized having *bla*_*KPC*_ gene, the pKpQIL plasmid and the Tn4401a transposon were evaluated, including 34 positive controls carbapenemase-producing *Klebsiella pneumoniae* associated with Tn4401a, 30 Enterobacterales *bla*_KPC_ positive of unknown plasmid background, and 146 negative controls. Accuracy and agreement were established for Vitek MS, RAPIDEC^®^ CARBA NP, and mCIM/eCIM) tests; ROC curves were compared among these tests.

**Results:**

The 11,109 Da peak was detected in 100% of KPC Tn4401a positive isolates using Vitek MS, sensitivity of 100% (95% CI 98.53–100), specificity of 95.5% (95% CI 91.7–99.4), positive predictive value (PPV) of 85.0 (95% CI 72.7–97.3), negative predictive value (NPV) of 100% (95% CI 99.6–100) and positive Likelihood Ratio (PLR) of 22.3 (10.2–48.8). Agreement between the three tests was 93.3% Kappa index of 0.90 (95% CI 0.83–0.97, *p* ≤ 0.05). ROC curves showed areas under the curve (AUCs) of 0.95, 0.96 and 0.96 for the VITEK MS, RAPIDEC CARBA NP and the mCIM/eCIM tests, respectively.

**Conclusion:**

Detection of the 11,109 Da peak by Vitek MS confirms the presence of KPC-type carbapenemase, allowing rapid and simultaneous detection with species identification; a negative result does not rule out the presence of the enzyme and may require additional tests.

**Supplementary Information:**

The online version contains supplementary material available at 10.1007/s10096-025-05097-6.

## Introduction

The emergence and spread of carbapenemase-producing *Enterobacterales* (CPE) have become a public health threat for the prevention, management, and control of infectious diseases [1, 2]. Their rapid dissemination, difficult control, and limited therapeutic options are associated with increased morbidity and mortality rates, and a high health care costs in patients infected by these microorganisms [3].

KPC-type carbapenemases (*Klebsiella pneumoniae* carbapenemases) are enzymes with a rapid dissemination capacity and now endemic in many countries. The first KPC producing bacteria was identified in North Carolina in a *Klebsiella pneumoniae* isolate [4, 5]. Since then, carbapenem resistant bacteria containing the *bla*_kpc_ gene expanded to the northeast of the United States, causing outbreaks in hospitals across New York and New Jersey, and then spread internationally to Puerto Rico, Greece, Italy, Poland, Israel, Eastern China, Argentina, Brazil, and Colombia [6, 7] In most of these countries, KPC-producing bacteria are currently considered endemic [8].

In Colombia, genome sequencing studies have demonstrated the presence of KPC-2 and KPC-3 producing *K. pneumoniae* in several sequence-types, and also has been reported in *Escherichia coli*, other Gram-negative bacilli and, in non-fermenting microorganisms such as *Pseudomonas aeruginosa* [2].

Successful dissemination of KPC-producing Enterobacteriacea is related to the *bla*_KPC_ gene, which is associated with the Tn4401 transposon included in IncFII plasmids; among these, pKpQIL is the most common plasmid vehicle for *bla*_KPC_ gene transmission [9]. Using technologies that allow the rapid detection of these elements and their association with the production of carbapenemases is crucial to guide timely and effective treatment, improving the chances of survival for patients and the effective control of in-hospital dissemination of carbapenemase-producing bacteria [10].

Matrix-assisted laser desorption ionization–time of flight mass spectrometry (MALDI-TOF MS) has been successfully incorporated in the microbiology laboratory setting, offering a fast and reliable alternative for identifying microorganisms [11, 12]. However, MALDI-TOF MS is not routinely used to detect resistance markers, as the molecular weight of the carbapenamase enzymes places them outside of the weight detection range routinely measured for the identification of bacterial species [13, 14].

Indirect detection of carbapenemases has been described for the detection of carbapenemase enzymes by monitoring and analyzing mass spectra of intact antibiotics versus the degraded products after exposure and hydrolysis by living carbapenemase-producing bacteria or, alternatively, using a direct detection of a characteristic peak pattern related to resistance determinants [15]. Several studies evaluating the indirect detection method have described the correspondence between the production of KPC-type carbapenemases and the presence of a peak of 11,109 Da corresponding to the P019 protein in the mass spectrum generated by MALDI-TOF MS for microorganism identification [16–18]. The p019 gene is associated with the Tn4401a transposon isoform as part of an insertion sequence frequently present in different plasmids harboring the blaKPC gene [16].

Few studies have evaluated the performance of the indirect detection method, identifying the 11.109 Da peak using MALDI-TOF Vitek MS. These studies used a small number of isolates from two healthcare institutions without ruling out the potential clonality of isolates, which may affect the actual performance of the test and its applicability in different epidemiological settings [19, 20].

The present study determined the diagnostic performance of the MALDI-TOF VITEK MS system in the detection of KPC-type carbapenemases through the direct identification of the characteristic peak 11,109 Da using a collection of carbapenem-resistant and non-resistant Enterobacterales from several health care institutions in Medellín, Colombia. In addition, we evaluated the agreement between the indirect MALDI-TOF detection method and other phenotypic tests conventionally used to detect KPC-type carbapenemases.

## Materials and methods

This was a diagnostic test performance study that aimed to validate the MALDI-TOF indirect detection method for the detection of KPC-type carbapenemases using isolates in which the *bla*_*kpc*_ gene, Tn4401a transposon and pKpQIL plasmids was confirmed by nucleic amplification methods (PCR-polymerase chain reaction) as the standard method. The study site was Laboratorio Medico de Referencia at Clínica El Rosario in Medellín, Colombia.

### Bacterial isolates

**Fig. 1:**
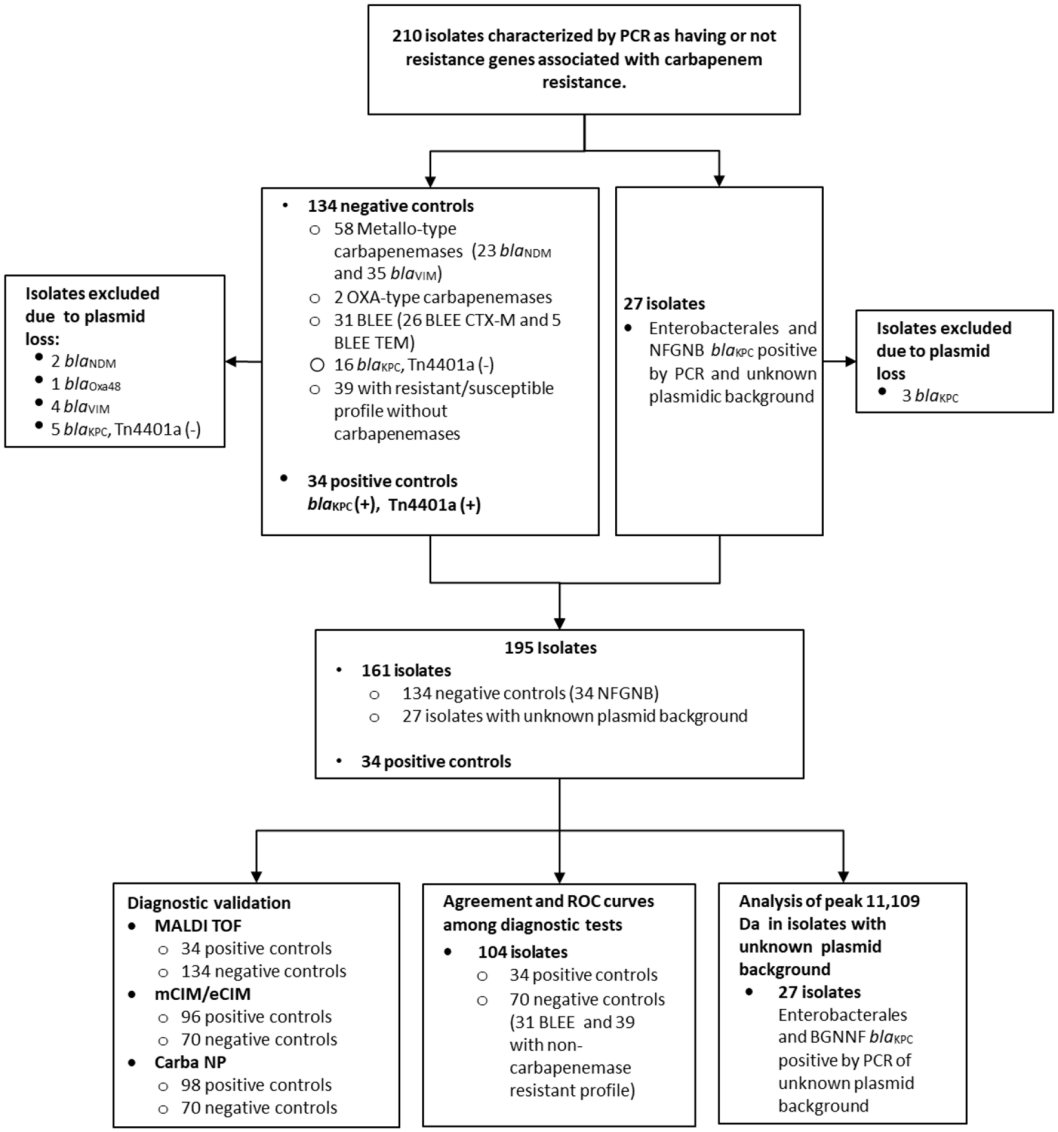
Characterization of isolates used in the study. From a total of 210 initial isolates, 15 isolates were excluded due to plasmid loss for a total of 195 microorganisms analyzed

We used a collection of 210 microorganisms preserved by the microbiology laboratory MICROBA at Universidad de Antioquia, isolated from 2012 until 2015, from hospitalized patients in 5 healthcare institutions in Medellin, Colombia. The collection of isolates was previously characterized using a PCR technique for the identification of carbapenemase genes according to the protocol developed by Poirel et al. [21], and using the protocol developed by Chen et al. for the detection of plasmids similar to pKpQIL and the Tn4401a transposon [22]. We selected 34 positive control isolates consisting of carbapenemase-producing *Klebsiella pneumoniae* strains that were positive for the *bla*_KPC_ gene, the pKpQIL plasmid and the Tn4401a transposon and 146 negative controls including other species of Enterobacterales and non-fermenting Gram-negative bacilli (NFGNB) carrying different resistance mechanisms, KPC-type carbapenemases not associated with the pKpQIL plasmid or the Tn4401a transposon and carbapenemases other than KPC (Fig. 1).

### Bacterial identification and antimicrobial susceptibility

The isolates stored at -70 °C were cultured twice on blood agar for 18–24 h at 35 °C and reidentified using the MALDI-TOF VITEK MS system system (bioMérieux, Marcy- l’Étoile, France) before inclusion in the study. Carbapenemase production confirmation was performed in all isolates by checking the antibiotic susceptibility profile of the isolates using the VITEK^®^ 2 automated system (bioMérieux, Marcy-l’Étoile, France), phenotypic carbapenemase detection using RAPIDEC^®^ CARBA NP (bioMérieux, Marcy l’Étoile, France) and mCIM in conjunction with eCIM methods [23]. Antibiotic susceptibility was interpreted according to The Clinical and Laboratory Standards Institute (CLSI) M100 Manual version 32 [23]. An isolate was considered resistant to carbapenems when an intermediate or resistant result to any carbapenems (ertapenem, imipenem, or meropenem) was observed [23]. Isolates previously characterized by molecular tests as carrying genes associated with carbapenemase production, which presented a carbapenemase-susceptible profile with negative phenotypic tests for carbapenemase detection were excluded from the study. The susceptibility profile for carbapenems was compared with the genetic characteristics of resistance and carbapenemase production. Susceptibility was also performed in KPC-type carbapenemase-producing isolates to other antibiotics such as ceftazidime/avibactam and aztreonam.

### Identification of the 11,109 Da peak by the indirect MALDI-TOF detection method

Using the default instrument parameters, the MALDI-TOF VITEK MS system was used in its two configurations, RUO and IVD, in a positive linear mode in the mass range of 2,000–20,000 *m/z*. The results were analyzed using the Saramis™ software (Spectral File and Microbe Identification System) (Version 4.10, AnagnosTec, Potsdam, Germany) and Shimadzu Biotech Launchpad^®^ software (Shimadzu Corporation, Kyoto, Japan) [19].

Data analysis was performed according to the manufacturer’s instructions as follows: the identification of the microorganism at the species level was considered valid in percentages between 75% and 99.9%, using *Escherichia coli* ATCC^®^ 8739 as a run control strain. The 11,109 Da peak was detected by visual inspection of the spectra obtained by MALDI-TOF VITEK MS using Saramis software, and a record of the corresponding spectrum was made. The 11,109 Da peak was considered positive in a range of 10 Da above or below this value (11,099 Da -11,119 Da). *K. pneumoniae* ATCC BAA 1705 (*bla*_*KPC*_-positive) and *K. pneumoniae* ATCC BAA 1706 (*bla*_KPC_-negative) strains were used as positive and negative controls for the presence of the KPC enzyme, respectively.

### Diagnostic accuracy of the indirect MALDI-TOF detection method for the detection of pKpQIL-borne KPC carbapenemases

An initial standardization using 30 isolates characterized as positive for the *bla*_KPC_ gene carrying the Tn4401a transposon was performed to determine the best quality of the spectra and the best method for sample preparation before dispensing the sample in the slide well for detection of the 11.109 Da peak. For this purpose, three procedures were compared: a direct method consisting in a direct deposition of the colony in the well, a formic acid method that consisted of the direct deposition of the colony in the well plus 1 µL of formic acid, and a sample extraction method as described by Rocco et al. consisting of an initial washing of the colonies with 70% alcohol, followed by protein extraction using formic acid and acetonitrile [19]. The respective mass spectra generated with the three methods were compared using the Vitek MS RUO and Vitek MS IVD systems.

The diagnostic accuracy of the indirect detection of carbepenemase by MALDI-TOF was evaluated using 34 positive controls (*bla*_KPC_, Tn4401a (+)) and 134 negative controls (11 *bla*_KPC_, Tn4401a (-),31 *bla*_VIM_, 21 *bla*_NDM_, 1 *bla*_OXA−51_*(+)*,* bla*_OXA−23_, 70 BLEE positive and Resistant/Susceptible to carbapenems without carbapenemases). Thirty KPC-producing isolates with an unknown plasmid background were also processed using the indirect MALDI-TOF detection method to identify the 11,109 Da peak when the presence of the Tn4401a transposon was unknown.

Diagnostic performance for the detection of carbapenemases by conventional methods such as RAPIDEC CARBA NP (bioMérieux, Marcy l’Etoile, France) and the mCIM method in conjunction with the eCIM method were performed using 98 (45 *bla*_KPC_ Tn4401a (+)/(-), 31 *bla*_VIM_, 21 *bla*_NDM_, 1 *bla*_OXA− 51_*(+)*,* bla*_OXA− 23_*(+)*) and 96 (45 *bla*_KPC_ Tn4401a (+)/(-), 31 VIM, 19 NDM, 1 *bla*_OXA− 51_ (+), *bla*_OXA− 23_*(+)*) positive controls respectively, and 70 negative (*BLEE* positive and Resistant/Susceptible to carbapenems without carbapenemases) controls for both methods and compared with MALDI-TOF VITEK^®^ MS (bioMérieux, Marcy l’Etoile, France) results.

The RAPIDEC CARBA NP technique was used according to the manufacturer’s instructions [24]. The results were read by three observers blinded to the results obtained with other methods of carbapenemase detection. Disagreements were solved in favor of the other two methods. The mCIM in conjunction with eCIM methods followed the standard method described by CLSI [23].

In addition, concordance, and agreement between MALDI TOF VITEK MS, RAPIDEC CARBA NP and mCIM in conjunction with eCIM were established using the 34 KPC-producing isolates (Tn4401a positive) as positive controls and 70 negative controls (*BLEE* positive and Resistant/Susceptible to carbapenems without carbapenemases). For this analysis, all carbapenemase-producing isolates other than KPC associated with Tn4401a were excluded due to the inability of MALDI TOF to detect them.

### Statistical analysis

Categorical variables were described in relative and absolute frequencies, and quantitative variables were described by reporting the mean and standard deviation or medians with their interquartile range according to the data distribution. For diagnostic accuracy, sensitivity and specificity values were calculated, as well as positive and negative predictive values and likelihood ratios with 95% confidence intervals.

Percent agreement and nominal Cohen’s Kappa coefficient (95% CI) were calculated to establish agreement between the three tests. For each test, a ROC curve was calculated, and a subsequent comparison was performed. Values of *p* ≤ 0.05 were considered statistically significant. Statistical analyses were performed using EPIDAT 4.2 [25].

## Results

From the 210 initial isolates, 15 were excluded from the study due to possible plasmid loss confirmed by negative phenotypic tests for carbapenemase detection and an antimicrobial susceptibility test showing susceptibility to carbapenems, leaving a total of 195 isolates for analysis (Fig. 1). A total of 168 strains were studied with the indirect MALDI-TOF detection method characterized as follows: *Klebsiella pneumoniaen* = 80 (47.6%), *Escherichia colin* = 29 (17.3%), *Enterobacter cloacaen* = 27 (16.1%), *Pseudomonas aeruginosan* = 27 (16.1%) and *n* = 5 (3%) of other gram-negative bacterial species. The previously known resistance mechanisms associated with the different species and detection of carbapenemases using the indirect MALDI-TOF detection method, mCIM/eCIM inactivation tests, and CARBA-NP are described in Table 1.

**Table 1:** Detection of carbapenemases using MALDI TOF VITEK MS (11099–11119 peak), mCIM/eCIM inactivation tests, and CARBA-NP, according to microorganism species, resistance genetic characteristics, and carbapenem susceptibility

Resistant genetic characteristics and phenotypic carbapenem susceptibility	*n* (168)	MALDI-TOF VITEK MS (11099–11119 peak detection)		mCIM/eCIM inactivation test		Rapidec Carba NP	
		Positive	Negative	Positive	Negative	Positive	Negative
**Serine-carbapenemases**							
*K. pneumoniae****bla***_**KPC**_, **Tn4401a (+)**	34	34	0	34	0	34	0
*K. pneumoniae****bla***_**KPC**_, **Tn4401a (-)**	11	1	10	11	0	11	0
**Metallo-β-lactamase**							
*P. aeruginosa* ***bla***_**VIM**_	27	1	26	16	9	27	0
*E. cloacae* ***bla***_**VIM**_	2	0	2	2	0	2	0
*P. stuartii* ***bla***_**VIM**_	1	0	1	1	0	1	0
*K. pneumoniae* ***bla***_**VIM**_	1	0	1	1	0	1	0
*K. pneumoniae* ***bla***_**NDM**_	14	1	13	14	0	14	0
*E. cloacae* ***bla***_**NDM**_	3	0	3	3	0	3	0
*K. oxytoca* ***bla***_**NDM**_	2	0	2	2	0	2	0
*C. freundii* ***bla***_**NDM**_	1	0	1	1	0	1	0
*A. baumanii* complex ***bla***_**NDM**_	1	0	1	0	1	1	0
*K. pneumoniae bla*_OXA−51_***(+)***, *bla*_OXA−23_***(+)***	1	0	1	1	0	1	0
***BLEE positive***							
*E. coli* **CTX-M**	24	2	22	0	24	1	23
*K. pneumoniae* **CTX-M**	2	0	2	1	1	1	1
*E. coli* **TEM**	5	0	5	0	5	0	5
Susceptible to carbapenems without carbapenemases*							
*Enterobacter cloacae*	11	0	11	0	11	0	11
*Klebsiella pneumoniae*	10	1	9	0	10	0	10
Resistant to carbapenems without carbapenemases**							
*Enterobacter cloacae*	11	1	11	1	10	1	10
*Klebsiella pneumoniae*	**7**	0	7	0	7	0	7

All isolates were confirmed by PCR for the presence of genes involved in Carbapenemase production

*With carbapenemase-free sensitive profile, ** With carbapenemase-free resistant profile

After the initial evaluation of methods for processing the samples and the systems for generating the best spectrum obtained by the MALDI TOF VITEK MS, the IVD system, and the direct method for preparing the sample were chosen. Using these methods, 34/34 (100%) bacteria carrying the *bla*_KPC_ (+) gene and Tn*4401a* had a detection of the 11.109 Da peak and 7/134 (5%) of the negative controls. The inactivation methods and CARBA NP were also positive in 34/34 (100%) of bacteria carrying the *bla*_KPC_ (+) gene and Tn4401a (Fig. 1S). In addition, the latter methods were positive for detecting carbapenemases in isolates presenting the *bla*_VIM,_*bla*_NDM_, *bla*_*OXA51*_, and *bla*_*OXA23*_ genes, except for mCIM/eCIM inactivation methods that failed to identify carbapenemases in 9 out of 27 *P. aeruginosa bla*_VIM_ (Table 1).

For the indirect MALDI-TOF detection method, the sensitivity and specificity were 100% (95% CI 98.5–100) and 95.5 (95% CI 91.7–99.4) respectively, with a PPV of 85.0 (95% CI 72.7–97.3) and NPV of 100 (95% CI 99.6–100). In addition, for each false positive, 22 true positives were identified (95% CI 10.2–48.8). The inactivation tests for carbapenems mCIM/eCIM and RAPIDEC CARBA NP showed a sensitivity of 89.58% (95%CI 82.95–96.21) and 100% (CI 99.49–100), and a specificity of 97.14% (95% CI 92.53–100) and 95.71% (95% CI 90.26–100), respectively (Table 2).

**Table 2:** Diagnostic accuracy of the indirect MALDI-TOF detection method, inactivation methods, and CARBA NP test for KPC-type carbapenemases detection, using reference isolates according to carbapenem resistance genes

Parameters	Indirect MALDI-TOF detection method*		Inactivation mCIM/eCIM		CARBA NP	
	*n* (168)	Value (95% CI)	*n* (166)	Value (95% CI)	*n* (168)	Value (95% CI)
Sensitivity	34/34	100 (98.5–100)	86/96	89.58 (82.9–96.2)	98/98	100 (99.4–100)
Specificity	128/134	95.5 (91.7–99.4)	68/70	97.14 (92.5–100)	67/70	95,7 (90.2–100)
PPV	34/40	85.0 (72.7–97,3)	86/88	97.73 (94.5–100)	98/101	97.3 (94.0-100)
NPV	128/128	100 (99.6–100)	68/78	87.19 (79.1–95.2)	67/67	100 (99.2–100)
PLR	-	22.3 (10.2–48.8)	-	31.35 (7.9-123.1)	-	25.0 (7.7–70.5)

* For detection of KPC-type carbapenemases (carbapenemase-producing *Klebsiella pneumoniae* strains that were positive for the *bla*_KPC_ gene, the pKpQIL plasmid, and the Tn4401a transposon) PPV: Positive Predictive Value, NPV: Negative Predictive Value, PLR +: positive Likelihood Ratio, PLR-: negative Likelihood Ratio, CI 95%: 95% confidence interval

**Fig. 2:**
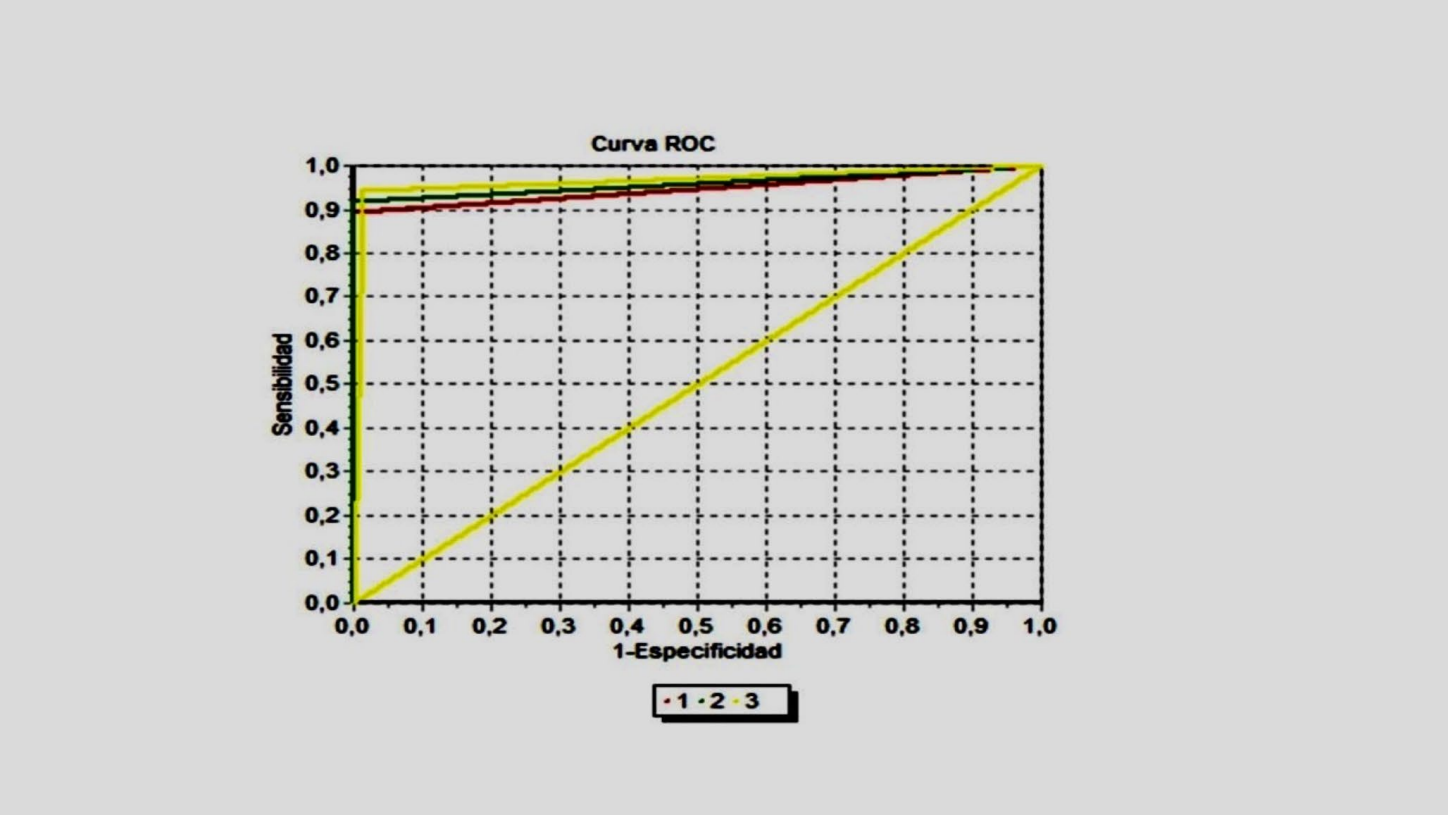
Comparison of the ROC curves of MALDI TOF Vitek MS, mCIM/eCIM inactivation methods and Rapidec CARBA NP methods for the detection of carbapenemases. (1) MALDITOF AUC = 0.95 (CI: 0.90–0.99), (2) CARB-NP AUC = 0.96 (CI: 0.91-1.00), (3) Inactivation AUC = 0.96 (CI: 0.92–1.01). p-value = 0.61

The agreement between the three diagnostic tests (evaluated in 104 isolates, 34 KPC, 31 BLEE and 39 Enterobacterales nonproducers of carbapenemases) was 93.3% with a Kappa index of 0.90 (CI 0.83–0.97, p-value: ≤ 0.05). No significant difference was found after comparing the ROC curves for each test (*p* > 0.05). The AUCs were 0.95 (CI: 0.90–0.99) for MALDI-TOF Vitek MS, 0.96 (CI: 0.92–1.01) for mCIM/eCIM inactivation method, and 0.96 (CI: 0.91 -1.00) for RAPIDEC CARBA NP (Fig. 2).

One hundred and sixty-eight isolates were evaluated using RAPIDEC CARBA NP by three observers blinded to the baseline results of carbapenemase production; a discrepancy between observers was observed in 10 (5,9%) results, yielding a Kappa of 0.91 (CI 0.85–0.96; *p* ≤ 0.05). These discrepancies occurred in non-carbapenemase-producing isolates, 8 ESBL-producing isolates, and two carbapenem-resistant but non-carbapenemase-producing isolates. In all cases, one of the three observers erroneously qualified the result as positive.

The susceptibility profile of the isolates studied showed that all *bla*_KPC_ Tn4401a (+)/Tn4401a (-), *bla*_NDM,_*bla*_VIM,_ and *bla*_OXA_ isolates presented a resistant or intermediate susceptibility profile for all three carbapenems evaluated (Table 1S). In addition, all isolates where the 11,109 Da peak was detected, had meropenem, ertapenem, and imipenem CIM of > = 16 µg/mL > = 8 µg/mL and > = 16 µg/mL, respectively.

Among the KPC-type carbapenemase-producing bacteria, 41/45 (91%) were susceptible to ceftazidime/avibactam; the only Oxa-type carbapenemase isolate was also susceptible, and 45/52 (87%) of metalo-carbapenemase-producing isolates showed a resistance profile to this antibiotic. For aztreonam, 44/45 (98%) of the KPC-type carbapenemase isolates showed a resistant profile; 25/52 (49%) of isolates producing metallo-carbapenemases were resistant, 9/52 (18%) had a susceptible profile, and 17/52 (33%) had an intermediate result.

## Discussion

The worldwide dissemination of KPC-type carbapenemase-producing Enterobacterales requires rapid identification methods for timely decision-making in critically ill patients. the indirect MALDI-TOF detection method through the detection of the 11,109 Da peak provides a rapid option for identifying the presence of these enzymes.

This study confirmed that MALDI TOF VITEK MS provides quick results due to the joint species identification of the microorganism and the 11,109 Da peak in the generated spectrum, indicating the indirect presence of KPC-type carbapenemase-producing microorganisms associated with Tn4401a. Thus, the applicability of this methodology may be dependent of the epidemiology and the genetic background of the KPC producing isolates in a specific setting. This rapid result should directly impact therapeutic decision-making for the initial management of patients, decreasing the time of hospitalization with significant savings in time and costs of diagnosis [3, 10].

The MALDI TOF VITEK MS diagnostic performance for identifying KPC found in the present study showed a sensitivity for the indirect MALDI-TOF detection method similar to values reported in other studies describing sensitivities ranging from 85.1 to 98.7% and specificities ranging from 99 to 100% [17, 19, 20, 26, 27]. The advantage of the present study, compared to others that have evaluated the indirect MALDI-TOF VITEK MS detection method, is that the isolates used were from different institutions, decreasing the possibility of a bias effect due to the potential clonality of nosocomial isolates from a single institution [19, 20].

In this study, seven isolates that were negative for KPC were identified as positive by the indirect MALDI TOF method. False positives may be explained by the presence of a protein of similar molecular weight as P019 with an analogous peak in the spectrum. In addition, this finding could also be explained by the independence in mobilization demonstrated between the tn4401a transposon, the ISKpn31 sequence and its possible presence in plasmids not related to the presence of KPC-type enzymes (an aspect that should be verified by whole genome sequencing analysis) [28, 29]. Several studies in Colombia have described the co-circulation of *bla*_KPC_ genes associated with Tn4401 transposon isoforms a and b and with elements other than Tn4401 [8, 30]. This scenario suggests that identifying the presence or absence of the 11,109 Da peak in the MALDI-TOF MS spectrum is helpful according to the epidemiological setting of the *bla*_KPC_ gene associated with the Tn4401a transposon. In the case of settings where other mechanisms mediate resistance to carbapenems or there is a circulation of different transposons carrying *bla*_KPC_, the presence of the 11,109 Da peak by MALDI TOF Vitek MS can confirm the presence of pKpQIL KPC-type carbapenemases, with a high positive predictive value, however, a negative result does not rule out resistance to carbapenems, and additional methods for detecting carbapenemases are needed.

The evaluation of the diagnostic performance for the mCIM/eCIM carbapenem inactivation test showed a sensitivity of 89.58% and a specificity of 97.14%, like those reported for other studies [31]. Although this technique has the benefit of differentiating metallo-carbapenemases and is less expensive [23], it is laborious and slow, involving a 4-hour incubation time during the inactivation step and a subsequent overnight incubation.

Rapidec CARBA NP also showed high sensitivity and specificity values (100% and 95.7%, respectively), like those described in other studies [31]. These performance values, together with the rapid identification of carbapenemase-producing bacteria, make this test a good option in the clinical setting for an initial approach to carbapenemase identification; however, since this technique does not differentiate the type of enzyme, it may require the use of additional discriminatory tests, especially in settings like Colombia where both metallo-carbapenemase and serine carbapenemases are endemic. In addition, when using this test, it is necessary to consider that low sensitivity for OXA48-type carbapenemase identification has been reported, and its visual interpretation, based on color variation, may have a degree of subjectivity [31, 32]. OXA-48-producing Enterobacteriaceae have a very low prevalence in Colombia but are endemic in other setting. In our study, in 10 out of 168 isolates, there was no agreement between the three observers when using RAPIDEC CARBA NP, and the kappa index was 0.91.

The comparison of the three methods used for the detection of the production of KPC-type carbapenemase showed good agreement and a similar AUC of the ROC curves. For rational use and considering the characteristics of the evaluated tests, a diagnostic algorithm may be implemented to establish the presence of carbapenemase-producing isolates. This algorithm should consider an early screening test to be the indirect MALDI TOF detection method, which allows the detection of the 11,109 Da peak together with the identification of the bacteria at no additional cost in an average time to result of 30 min, when a positive result is obtained, the presence of a KPC-type carbapenemase can be confirmed and reported with a high positive predictive value. Due to the low negative predictive value, a negative result by MALDI-TOF should be followed by performing the RAPIDEC CARBA NP test, which allows confirmation of carbapenemase presence or absence in a maximum time of 2 h. A negative result rules out the presence of any enzyme, but a positive result should be followed by eCIM/mCIM tests to differentiate metallo-type carbapenemases from other carbapenemases or by a rapid carbapenemase detection methods such as those based on immunochromatography or molecular methods [33].

According to the susceptibility profile, all carbapenemase-producing bacteria and, in particular, the KPC-type bacteria carrying the Tn4401a transposon were resistant to all carbapenems: meropenem, ertapenem, and imipenem [23]. As expected, most of the KPC and OXA carbapenemase-producing isolates evaluated were susceptible to ceftazidime/avibactam and aztreonam [34]. Nevertheless, 9% of the isolates presented resistance to these drugs, suggesting the presence in our setting of additional resistance mechanisms, such as mutations in *amp*C, *bla*_KPC−3_ carriers with omega-loop mutations [35, 36], changes in PBPs, or increased efflux pumps [37]. Among the metallo carbapenemases-producing isolates evaluated, 13% presented an unusual profile of susceptibility to ceftazidime/avibactam, considering that the most common mechanism of acquired resistance to this antibiotic is the production of avibactam-refractory beta-lactamases such as class B enzymes [38]. On the other hand, a resistance profile was observed for aztreonam in most of the isolates carrying the *bla*_KPC_ gene, as expected. In contrast, the isolates producing metallo-carbapenemases showed a more heterogeneous profile, suggesting that metallo-carbapenemases do not hydrolyze aztreonam, and their action may be affected by the existence of additional serine-type beta-lactamases [39, 40].

The present study described and validated MALDI-TOF MS technology as a rapid and efficient method for the identification of KPC-producing Enterobacterales carrying the Tn4401a transposon; furthermore, its inclusion as part of a diagnostic algorithm would allow savings in time and costs by rapidly detecting KPC-type carbapenemases simultaneously with microbial identification. However, an important limitation of the method evaluated is its inability to detect carbapenemases other than those carrying by the Tn4401a transposon, a situation that is conditioned by the prevalence of the KPC gene associated with the *a* isoform of the Tn4401 transposon. Therefore, prior to the routine use of the methodology, it is important to know the epidemiology and genetic background of clinical isolates. Additionally, according to the timing of collection of the study strains (2012–2015) it is possible that they do not represent the current epidemiological situation in our setting with respect to the circulation of microorganisms and their resistance mechanisms.

In conclusion a positive result should be used as a first step for the indirect detection of KPC-producing associated to Tn4401a in Enterobacterales, while an additional confirmatory test must confirm negative results.

## Electronic supplementary material

Below is the link to the electronic supplementary material.

Supplementary Material 1

Supplementary Material 2

## Data Availability

All data generated or analyzed during this study is available upon request; send any request to jrobledo@labmedico.com.

## Abbreviations

KPC: Klebsiella pneumoniae carbapenemases
Da: Daltons
MS: Mass Spectrometry
PCR: Protein chain reaction
mCIM: Modified carbapenemase inactivation method
eCIM: EDTA-modified carbapenem inactivation method
CPE: Carbapenemase-producing Enterobacterales
NFGNB: Nonfermentative Gram-negative bacilli
CLSI: Clinical and Laboratory Standards Institute
PPV: Positive Predictive Value
NPV: Negative Predictive Value
PLR: Positive Likelihood Ratio
AUC: Area under the curve
ESBL: Extended expectrum beta lactamase
OXA: Oxacillinase
PBP: Penicillin binding proteins
