# Supplementary figures and images for: Use of MALDI-TOF VITEK MS for rapid and efficient identification of KPC-type carbapenemases in Enterobacterales carrying the Tn4401a transposon
Source: Eur J Clin Microbiol Infect Dis. 2025 Apr 3;44(6):1443–53. doi: 10.1007/s10096-025-05097-6 (PMC12116874; doi:10.1007/s10096-025-05097-6)

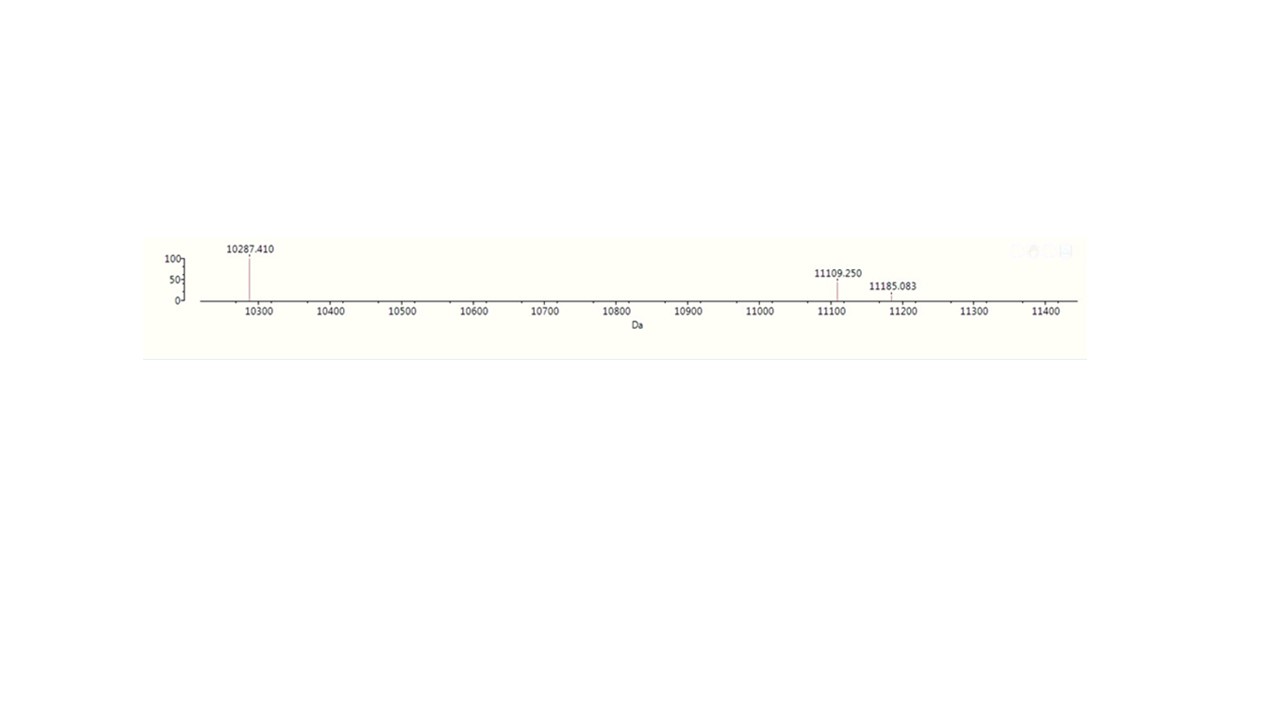

Supplement: Supplementary file 1 — Supplementary Material 1 [file 10096_2025_5097_MOESM1_ESM.jpg]
