# Supplementary material for: Use of MALDI-TOF VITEK MS for rapid and efficient identification of KPC-type carbapenemases in Enterobacterales carrying the Tn4401a transposon
Source: Eur J Clin Microbiol Infect Dis. 2025 Apr 3;44(6):1443–53. doi: 10.1007/s10096-025-05097-6 (PMC12116874; doi:10.1007/s10096-025-05097-6)
Supplement: Supplementary file 2 — Supplementary Material 2 [file 10096_2025_5097_MOESM2_ESM.docx]

**
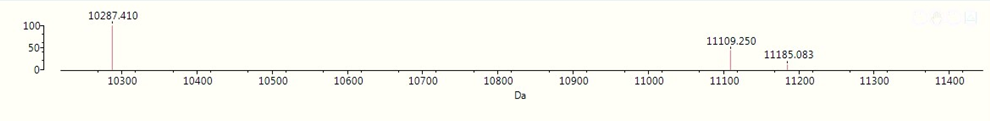
**

**Fig.1S Detection of the 11.109 Da peak in the Vitek ms mass spectrum.**

Detection of the 11,109 Da peak *in Klebsiella pneumoniae* carrying the blaKPC (+) gene and Tn4401a.

**Table 1S. Carbapenem susceptibility profile according to genetic characteristics of resistance of microorganisms used during the diagnostic accuracy description of the indirect MALDI-TOF detection method.**

| **Genetic Characteristics of resistance** | **Meropenem** | **Imipenem** | **Ertapenem** |
| --- | --- | --- | --- |
|  | **R/I/S*** | **R/I/S*** | **R/I/S*** |
| *bla*_KPC_, Tn4401a | 34/0/0 | 34/0/0 | 34/0/0 |
| *bla*_NDM_ | 21/0/0 | 20/1/0 | 20/0/0 |
| *bla*_VIM_ | 25/5/0 | 31/0/0 | 3/0/0 |
| *bla*_Oxa_ | 1/0/0 | 1/0/0 | 1/0/0 |
| *bla*_CTX-M_ | 1/0/25 | 1/0/25 | 1/0/25 |
| *bla*_TEM_ | 0/0/5 | 0/0/5 | 0/0/5 |
| *bla*-KPC, Tn4401a (-) | 11/0/0 | 11/0/0 | 8/1/0 |
| Susceptible to all carbapenems | 0/0/21 | 0/0/21 | 0/0/20 |
| Resistant to all carbapenems** | 3/1/14 | 5/2/11 | 8/4/2 |

*** R:** number of resistant isolates/I:number of intermediate isolates/S: number of susceptible isolates

**Resistant to all carbapenems, with resistance due to a different mechanism than carbapenamase production
